# Alleviation of masticatory disturbance with an occlusal splint in a Duchenne muscular dystrophy patient

**DOI:** 10.1111/scd.12594

**Published:** 2021-04-07

**Authors:** Yutaka Fukumoto, Takeshi Miyama

**Affiliations:** ^1^ Department of Dentistry, National Center Hospital National Center of Neurology and Psychiatry Kodaira Japan; ^2^ Department of Surgery National Center Hospital, National Center of Neurology and Psychiatry Kodaira Japan

**Keywords:** Duchenne, malocclusion, mastication, muscular dystrophy

## Abstract

**Aim:**

To present an occlusal splint effective for alleviating masticatory disturbance in Duchenne muscular dystrophy (DMD).

**Case report:**

A 13‐year‐old male DMD patient with masticatory disturbance presented with an open bite, with occlusal contact only between the first and second molars bilaterally and reduced masticatory performance. We applied an occlusal splint that achieved occlusal contact for all teeth and monitored its effects on masticatory function over 6 years. The occlusal splint increased occlusal contact points from 11 to 60. Although occlusal force remained at 13.9‒16 kg, masticatory performance increased, and the number of mastication strokes increased from 124 to 169. Masseter muscle activity decreased from 76.8% to 33.4% maximum voluntary contraction (MVC) and digastric muscle activity increased from 8.7% to 18.0% MVC. Time from start of peanut mastication to swallowing decreased, and the vertical mastication cycle diameter and its width on the habitual side increased.

**Conclusions:**

Masticatory disturbance in a DMD patient was alleviated using an occlusal splint. The number of mastication strokes and the digastric to masseter muscle activity ratio were increased. Furthermore, the mastication cycle was enlarged, which increased masticatory movement. As masseter muscle activity during mastication decreased, the occlusal splint likely reduced muscle fatigue during masticatory movement.

## 1 INTRODUCTION

Duchenne muscular dystrophy (DMD) is an X‐linked recessive disease caused by dystrophy gene mutations.^1^ DMD is an exceedingly rare disease, with a 2020 meta‐analysis reporting the birth prevalence of DMD as 19.8 per 100,000 live male births.^2^

Skeletal muscle fibers undergo necrosis, fibrosis, and conversion to fatty tissue, resulting in whole‐body muscular strength decrease and muscular atrophy.^3^

The masticatory muscles, particularly the masseter muscle, are also affected.^4^ The occlusal force of DMD patients is decreased to 8.1‒18.9 kg, as compared to 52.2 kg in healthy subjects.^5^, ^6^ Degeneration of the perioral muscles^7^ causes sagittal contraction and lateral expansion of the dental arch, protrusion of the maxillary incisors, retrusion of the mandibular incisors, lateral mandibular expansion beyond the maxilla, and abnormal facial morphology due to excessive vertical growth.^8^, ^9^ Consequently, DMD patients commonly experience malocclusion, particularly open bite,^8^, ^9^ extending from the anterior teeth to the molars.^10^ Their masticatory performance is reduced, due to a decrease in occlusal force and the number of occlusal contact points.^10^

Van Bruggen et al used chewing gum training,^11^ while Miller^12^ and Leinbach^13^ used orthodontic treatment and prosthetic treatment, respectively, to alleviate decreased masticatory performance in DMD patients. The use of surgical splints in edentulous and pediatric patients with maxillary or mandibular fractures^14^, ^15^ and occlusal splints (OSs) in patients with temporomandibular disorders, can alleviate symptoms by rapidly stabilizing the occlusal contact relationship^16^, ^17^ and be a viable alternative to time‐consuming prosthetic measures.^18^

We here report the case of a DMD patient with masticatory disturbance, which was effectively alleviated by treatment with an OS adjusted to achieve occlusal contact of all teeth.

## 2 CASE REPORT

A 13‐year‐old male patient with DMD presented with a primary complaint of stress when eating school lunches, because he was unable to bite through the fried *chikuwa* (baked tubes of reconstituted fish).

At 3 years of age, he had been unable to climb stairs or run and fell over easily. At 4 years, a right bicep muscle biopsy led to a diagnosis of DMD. By 8 years, walking had become difficult, and he started using an electric wheelchair. At the age of 10 years, he became aware of the masticatory disturbance, and his principal food at home had become rice porridge, with bite‐size side dishes precut for him.

At the initial examination, the patient utilized an electric wheelchair, which was operated with a button wheelchair joystick control. Although he required full care during meals, he showed no swallowing or respiratory function disorders.

**FIGURE 1:**
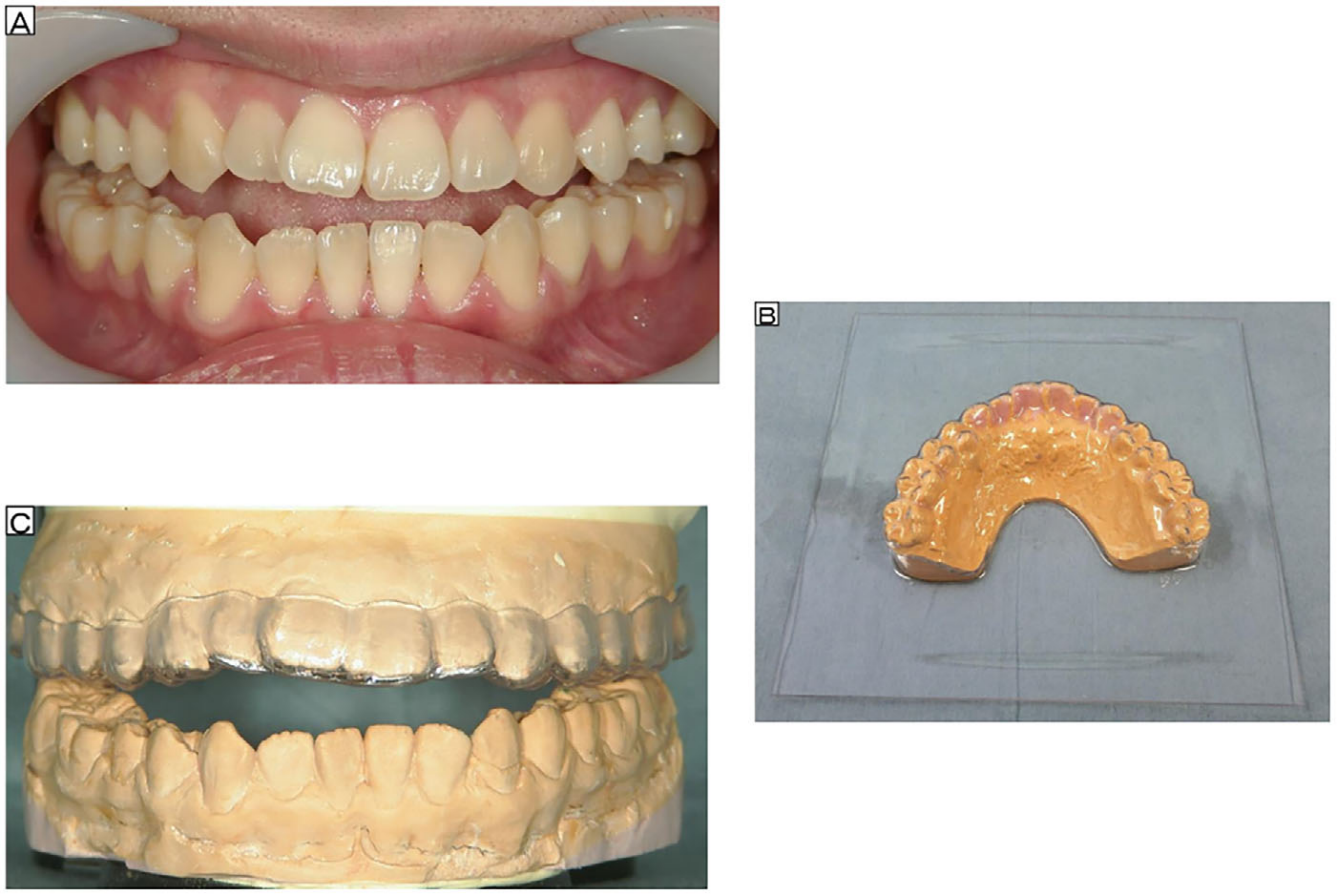
(A) Open bite, without occlusal contact, except at the first and second molars bilaterally. (B) Vacuum‐formed thermoplastic splints pressure‐welded into the maxillary model. (C) The occlusal splint is adjusted on maxillary and mandibular models mounted on an articulator to avoid changes in the original jaw position or vertical occlusal dimensions

None of his 28 teeth were missing; his oral hygiene status was favorable, and caries and periodontal disease were absent. The dental arch width of the mandible exceeded that of the maxilla, and he had bilateral open bite, with no occlusal contact other than for the first and second molars (Figure 1A). The maximum mouth opening distance was 60 mm, including an open bite distance of 4 mm in the incisor region, and jaw noise and pain were absent.

### 2.1 Treatment

The patient's mother was instructed regarding the necessity for careful oral hygiene to maintain his oral status after OS provision. Firstly, tooth models were prepared for the maxilla and mandible. For esthetic reasons, Splint‐a (Yamahachi Dental Manufacturing Co., Ltd., Aichi, Japan), a vacuum‐formed thermoplastic splint (1.5‐mm thick), was prepared by pressing into models using a Sta‐Vac (Buffalo Dental Manufacturing Co., Inc., New York, USA; Figure 1B).^14^

**FIGURE 2:**
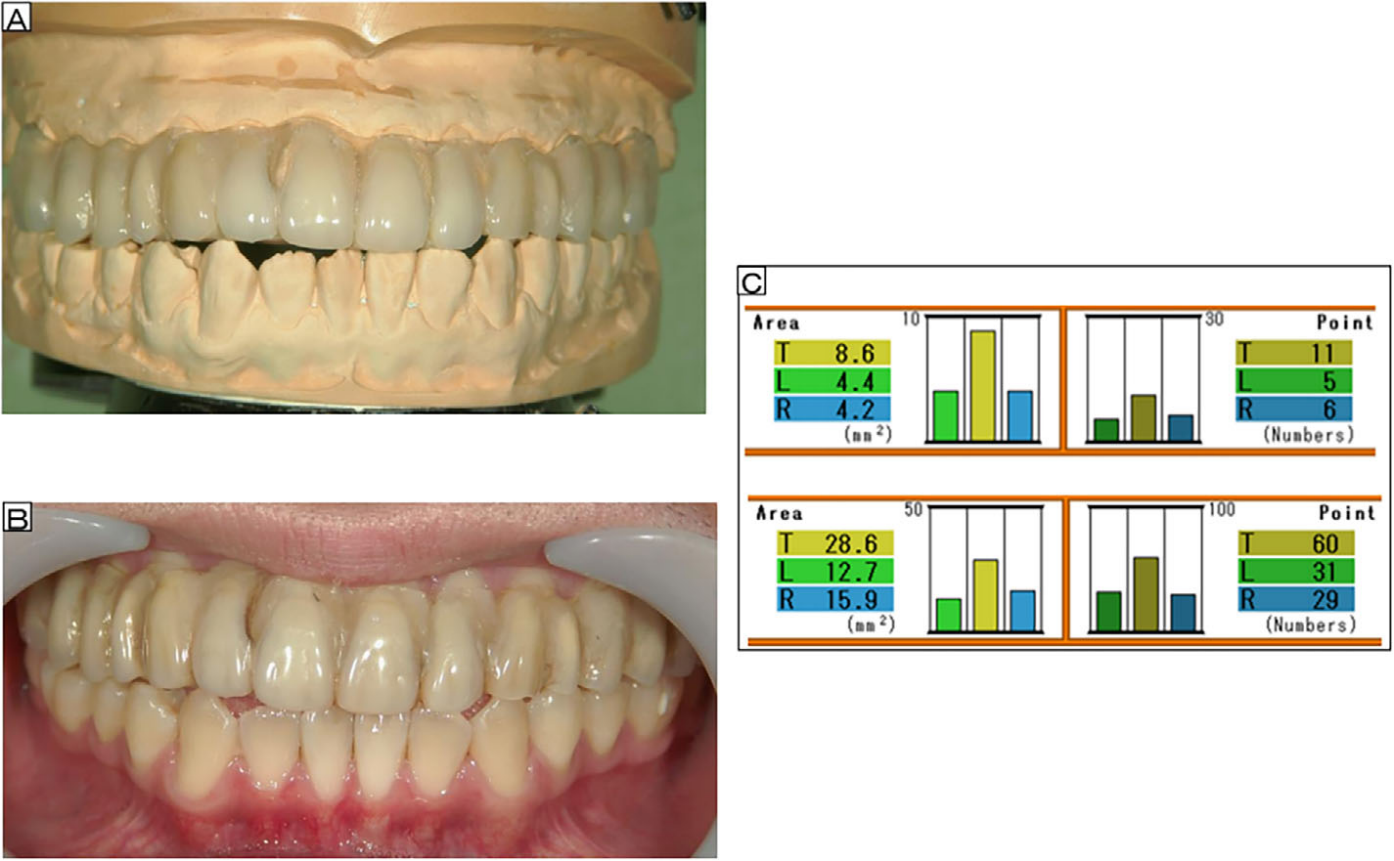
(A) The occlusal splint is adjusted on maxillary and mandibular models mounted on an articulator to achieve occlusal contact using self‐curing resin and abrasion‐resistant artificial teeth at the points without occlusal contact. (B) The occlusal splint is adjusted so that occlusal contact is achieved with all teeth in the opposing arch. (C) Results for the surface area and number of occlusal contact points, before and after use of the occlusal splint, as measured using the Biteye (GC Co., Ltd., Tokyo, Japan)

After discussion with the patient, a maxillary OS was fabricated to cause minimal discomfort to the tongue. A jaw position was selected with an occlusal relationship in which the patient felt that the teeth of both jaws were in contact and had maximum stability. Bite checking was performed, and maxillary and mandibular models were mounted on an articulator, such that the OS did not cover the existing occlusal contacts or cause changes in the original jaw relationship and occlusal vertical dimension (Figure 1C). Self‐curing resin and abrasion‐resistant artificial teeth (Endura; Shofu Co., Ltd., Kyoto, Japan) were added. Adjustments were made to ensure occlusal contact with the incisal edges of the opposing lower anterior teeth and the buccal cusps of the lower molars (Figures 2A and 2B), as well as to achieve group function on the working side during lateral excursion.

### 2.2 Progression

The effects of the OS on mastication were investigated based on the occlusal force and masticatory performance. Occlusal force was measured using an occlusal force meter (Nagano Keiki Co., Ltd., Nagano, Japan). The sensor was placed on the occlusal surface of the first maxillary molar on the habitual side, and measurements were made with the patient clenching his teeth three times, separated by 20‐s relaxation intervals. The highest of the three measurements was used in the analysis.^19^

Masticatory performance was determined using Masticatory Performance Evaluating Gum XYLITOL (Lotte Co., Ltd., Saitama, Japan),^20^ and the mastication of peanuts.^21^ The masticatory performance test with gum required the patient to chew the gum for 2 min on his habitual side; the masticatory performance, number of mastication strokes, and masseter and digastric muscle activities were then assessed. The masticatory performance was determined via a color change in the gum after mastication and judged to be higher when the color was closer to red, with reference to a color scale.^20^

Muscle activity was recorded by surface electromyogram, using an electrical stimulator with an attached Holter electromyograph ME3000 (Thought Technology, Ltd., Montreal, Canada). The triode disposable electrodes, at 2‐cm spacing, were placed on the habitual side in the anteroposterior central region for the masseter muscle, and along the anterior belly muscle path for the digastric muscle. The surface‐electromyographic signals were differentially amplified as follows: sensitivity, gain accuracy of ±0.5%; frequency, 2048 Hz; and input impedance, at least 1 MΩ. Full‐wave rectification was performed, and the data were input into a personal computer with 14‐bit AD conversion, and filter‐treated with a better/worse filter (3 dB at 500 Hz). The amplitude was then obtained as the actual value using the BioGraph Infiniti software (Thought Technology, Ltd., Montreal, Canada).

The number of mastication strokes was calculated from the amplitudes of the masseter muscle. The patient voluntarily clenched his teeth and maintained a maximum mouth opening three times for 5 s, with 20‐s relaxation intervals. The maximum amplitude of each time was measured, and the mean values were taken to be the amplitudes of maximum voluntary contraction (MVC) of the masseter and digastric muscles. The mean amplitude while masticating the gum was then obtained and normalized for the ratio of the mean amplitude of MVC (%MVC) for each muscle.^19^

The masticatory performance test with peanuts involved a comparison of the time required (from the initiation of mastication) for the swallowing of five peanuts, with and without the OS; a shorter duration indicated a greater masticatory performance.^21^

After the patient had used the OS for 6 years, the mastication cycles for gum were also compared with and without the OS.^4^, ^19^ Analysis of the mastication cycles was performed using Kinect (Microsoft Corp., Redmond, WA, USA).^22^ A specialized hemispherical reflection marker was fitted to the mentum region of the patient while he was seated in a wheelchair.^23^ The Kinect V2 sensor was fixed in position on a tripod, 70 cm in front of the patient^24^ and 115 cm above the floor. Using a three‐dimensional motion analysis system ICpro‐K2 system (Hu‐tech Co., Ltd., Tokyo, Japan), the track of the marker was obtained by following it for 12 s at 30 frames/s. Finally, after stabilization, five consecutive strokes with and without the OS were compared.^19^, ^25^

At the initial examination, when the patient was 13 years old, the occlusal force was 16.4 kg, and the gum color was pale pink following the masticatory performance test. The number of mastication strokes was 124, and the masseter muscle activity was 77.9% MVC (the digastric muscle activity was not measured). Using Biteye (GC Co., Ltd., Tokyo, Japan), the number of occlusal contact points was 11, and the occlusal contact area was 8.6 mm^2^.

The OS was fitted 2 months after the initial examination. Initially, the patient complained of an unpleasant oral sensation while using the OS, but 5 months later at the age of 14, he routinely used the OS when eating. Changes in his occlusion were observed during this period, with the number of occlusal contact points and occlusal contact area having increased to 60 and 28.6 mm^2^, respectively (Figure 2C). After 6 months of routine OS use, at the age of 14.5 years, the patient no longer felt stressed when eating school lunches.

At the age of 16 years, the patient could eat not only *chikuwa* during school lunches, but also side dishes, without needing them to be cut to size. Mastication was easier, even on the non‐habitual side. Subsequently, the left molar region of the OS was damaged, and the left maxillary canine and premolar artificial teeth were lost, requiring repairs. The patient then found it more difficult to eat without the OS, whereas with the OS he had no problems with any foods other than *mochi* (pounded sticky rice), which is highly adhesive. However, the patient then stopped visiting our department for treatment and was lost to follow‐up.

**FIGURE 3:**
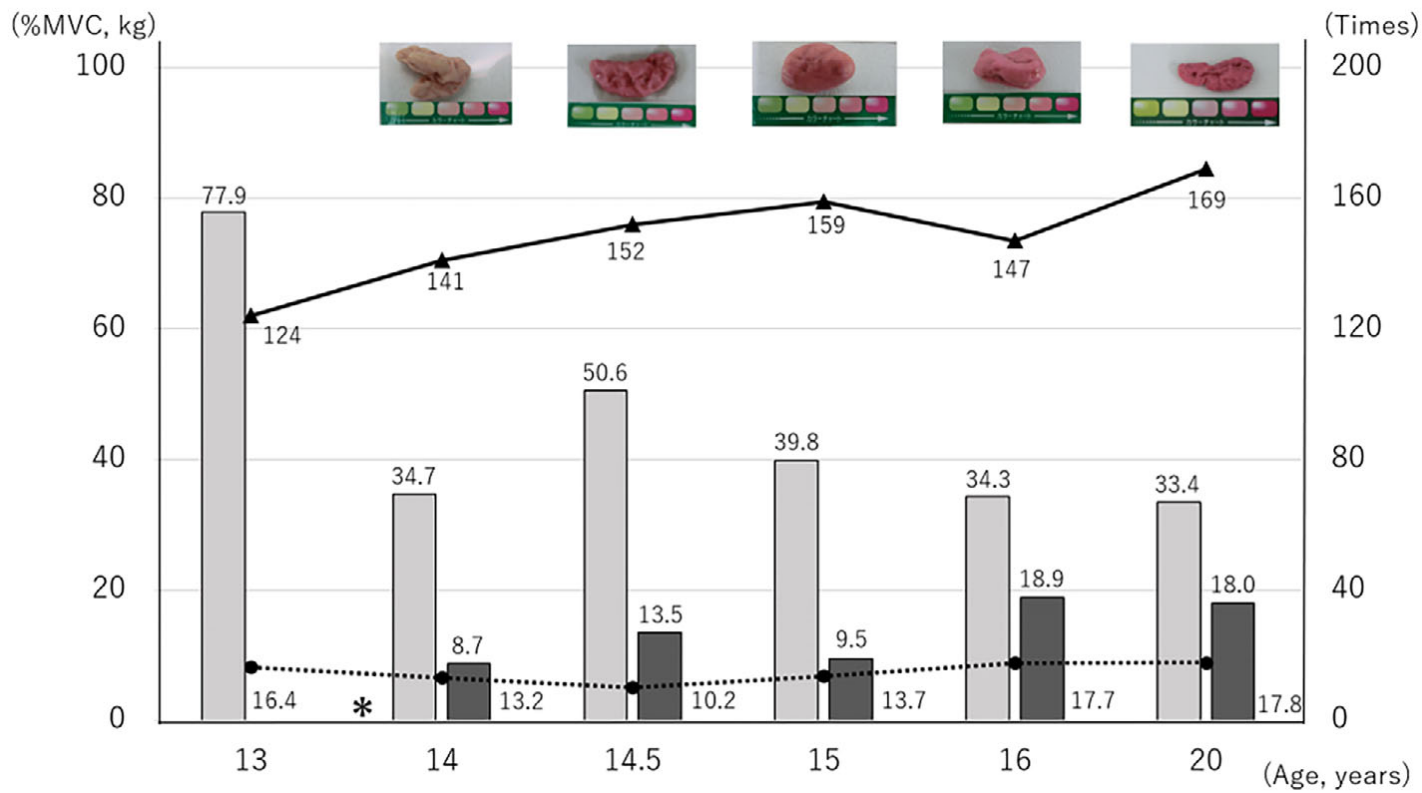
Progressive effects of the occlusal splint on mastication photos masticatory performance determined by the color‐changeable chewing gum. Normalized masseter muscle activity (% MVC: the percent ratio of the mean amplitude while masticating the gum divided by the amplitude of maximum voluntary contraction): Normalized digastric muscle activity (% MVC not determined at the age of 13 years). Solid line: Number of mastication strokes. Dotted line: Bite force (kg). ＊Occlusal splint setting

**FIGURE 4:**
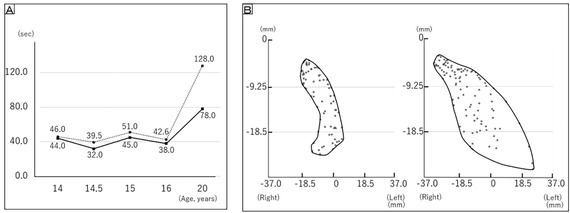
Comparison of effects on mastication with and without occlusal splints. (A) Time from start of mastication until swallowing of five peanuts. Dotted line: Occlusal splint not applied. Solid line: Occlusal splint applied. The time from the start of mastication until swallowing was reduced by use of the occlusal splint. (B) Mastication cycle with gum. Left: Occlusal splint not applied. Right: Occlusal splint applied. Use of the occlusal splint increased both the vertical diameter of the mastication cycle and its width in the direction of the working side (left)

At the age of 20 years, after routinely using the OS for 6 years, the patient experienced a decrease in respiratory function during sleep and required hospitalization for mechanical ventilation. The effects of the OS over the preceding 6 years were re‐examined by making comparisons with assessments conducted at the age of 14.5 years. The findings were as follows: the occlusal force remained below 18 kg; the gum color became dark pink; the number of mastication strokes increased from 152 to 169; masseter muscle activity decreased from 50.6 to 33.4% MVC; digastric muscle activity increased from 13.5 to 18.0% MVC; the absence of the OS increased the time to swallowing, during the masticatory performance test with peanuts, from 7.5 to 50 s; and the use of the OS during the mastication performance test with gum increased both the vertical diameter and the width of the mastication cycle in the direction of the working side (Figures 3 and 4).

## 3 DISCUSSION

In this case report, we showed that an OS can alleviate masticatory disturbance in a DMD patient. Van Bruggen et al investigated the effects of chewing gum training on the masticatory performance of DMD patients and found that, although occlusal force remained low, masticatory performance improved; the latter may have been attributed to the ability of such training to promote the skills required for the control and coordination of mandibular movements.^11^ However, the study's follow‐up did not exceed 1 month, and whether improvement was maintained is unclear.

Orthodontic treatment of malocclusion in DMD patients is difficult, and progressive development of craniofacial abnormalities is unpredictable.^26^ Miller reported that orthodontic treatment improved masticatory performance in DMD patients.^12^ However, they performed partial glossectomy for macroglossia to prevent the tongue from moving posteriorly post‐orthodontic treatment and tracheostomy to prevent postoperative asphyxiation. These treatments are highly invasive, resulted in respiratory infections which needed to be treated and led to the entire course of treatment taking as long as 21 months.^12^

Leinbach reported that a partial denture with overlays on the posterior teeth improved bilateral posterior open bite in a DMD patient.^13^ Nevertheless, this study did not show an actual improvement in masticatory performance. Notably, the partial denture had a metal framework with cast retentive clasps; in contrast, the OS used in the present study had a lower cost and required less laboratory time.

Masticatory performance improves with increasing occlusal force in the molar region, increased numbers of occlusal contact points, and greater vertical and horizontal mandibular movement during mastication.^27^ Therefore, we considered that increasing the number of occlusal contact points was necessary to alleviate masticatory disturbance in the present case. Additionally, owing to the patient's decrease in activities of daily living, the number of hospital visits and the invasiveness of the procedure needed to be minimized. Furthermore, it was pertinent that the newly established occlusal relationship did not give rise to temporomandibular joint disorder or cause esthetic concerns. Therefore, we chose to use an OS adjusted to enable occlusal contact by all teeth.

Bakke et al reported that the mastication cycle in MD patients with open bite was decreased by approximately 33% compared to healthy people.^4^ Moreover, Michler et al reported that if prosthetic treatment was used to restore occlusal contact points lost due to missing teeth, the mastication cycle increased in both the vertical and horizontal dimensions, particularly in the lateral direction on the working side, indicating improved masticatory performance.^25^ In this study, the OS increased the number of occlusal contact points and masticatory movement.

It has been reported that in patients with myotonic dystrophy, the occlusal force is decreased to 21% of that in healthy people. On the contrary, in terms of masticatory movement, the mean % MVC of the masticatory muscles is 40%, compared to 12.1% in healthy people.^19^ Indeed, masseter muscle activity may be increased to compensate for decreased masticatory function.^19^ Additionally, if the number of occlusal contact points at the intercuspal position increases (and the occlusal contact relationship is stabilized), masseter muscle contraction time and the imposed load decrease, thus lessening masseter muscle fatigue.^28^ Consequently, decreased masseter muscle % MVC with the OS was likely to have reduced the load on the masseter muscle of our patient during masticatory movement.

In patients with DMD, disproportionate morphological changes are first evident in the late mixed dentition or early permanent dentition,^8^ and may be associated with malocclusion, with more than one‐third of patients presenting with open bite.^29^ These changes may also present difficulties with bolus preparation and propulsion due to macroglossia, which become more prominent with advancing age.^29^ Further research is warranted to confirm whether the OS can prevent further tooth movement and whether it impairs tongue movement with aging. Future studies should consider evaluating OS effectiveness across different age groups.

In conclusion, the use of an OS improved masticatory movement and alleviated masticatory disturbance in a DMD patient by increasing the number of mastication strokes and the digastric to masseter muscle activity ratio and enlarging the mastication cycle.

## CONFLICT OF INTEREST

The authors declare that there is no conflict of interest that could be perceived as prejudicing the impartiality of the research reported.

## ETHICS STATEMENT

This manuscript is a case report in which the patient cannot be identified, and therefore the requirement for obtaining informed consent from the patient was waived. The work described has been carried out in accordance with The Code of Ethics of the World Medical Association (Declaration of Japan).
